# 20 years of the Bio-Analytic Resource for Plant Biology

**DOI:** 10.1093/nar/gkae920

**Published:** 2024-10-23

**Authors:** Alexander Sullivan, Michael N Lombardo, Asher Pasha, Vincent Lau, Jian Yun Zhuang, Ashley Christendat, Bruno Pereira, Tianhui Zhao, Youyang Li, Rachel Wong, Faisal Z Qureshi, Nicholas J Provart

**Affiliations:** Department of Cell and Systems Biology/Centre for the Analysis of Genome Evolution and Function, University of Toronto, 25 Willcocks Street, Toronto, ON M5S 3B2, Canada; Faculty of Science, University of Ontario Institute of Technology, 2000 Simcoe Street North, Oshawa ON L1G OC5, Canada; Department of Cell and Systems Biology/Centre for the Analysis of Genome Evolution and Function, University of Toronto, 25 Willcocks Street, Toronto, ON M5S 3B2, Canada; Department of Cell and Systems Biology/Centre for the Analysis of Genome Evolution and Function, University of Toronto, 25 Willcocks Street, Toronto, ON M5S 3B2, Canada; Department of Cell and Systems Biology/Centre for the Analysis of Genome Evolution and Function, University of Toronto, 25 Willcocks Street, Toronto, ON M5S 3B2, Canada; Department of Cell and Systems Biology/Centre for the Analysis of Genome Evolution and Function, University of Toronto, 25 Willcocks Street, Toronto, ON M5S 3B2, Canada; Department of Cell and Systems Biology/Centre for the Analysis of Genome Evolution and Function, University of Toronto, 25 Willcocks Street, Toronto, ON M5S 3B2, Canada; Department of Cell and Systems Biology/Centre for the Analysis of Genome Evolution and Function, University of Toronto, 25 Willcocks Street, Toronto, ON M5S 3B2, Canada; Department of Cell and Systems Biology/Centre for the Analysis of Genome Evolution and Function, University of Toronto, 25 Willcocks Street, Toronto, ON M5S 3B2, Canada; Department of Cell and Systems Biology/Centre for the Analysis of Genome Evolution and Function, University of Toronto, 25 Willcocks Street, Toronto, ON M5S 3B2, Canada; Faculty of Science, University of Ontario Institute of Technology, 2000 Simcoe Street North, Oshawa ON L1G OC5, Canada; Department of Cell and Systems Biology/Centre for the Analysis of Genome Evolution and Function, University of Toronto, 25 Willcocks Street, Toronto, ON M5S 3B2, Canada

## Abstract

The Bio-Analytic Resource for Plant Biology (‘the BAR’, at https://bar.utoronto.ca) is celebrating its 20th year in operation in 2025. The BAR encompasses and provides visualization tools for large ‘omics data sets from plants. The BAR covers data from Arabidopsis, tomato, wheat, barley and 29 other plant species (with data for 2 others to be released soon). These data include nucleotide and protein sequence data, gene expression data, protein-protein and protein–DNA interactions, protein structures, subcellular localizations, and polymorphisms. The data are stored in more than 200 relational databases holding 186 GB of data and are presented to the researchers via web apps. These web apps provide data analysis and visualization tools. Some of the most popular tools are eFP (‘electronic fluorescent pictograph’) Browsers, ePlants and ThaleMine (an Arabidopsis-specific instance of InterMine). The BAR was designated a Global Core Biodata Resource in 2023. Like other GCBRs, the BAR has excellent operational stability, provides access without login requirement, and provides an API for researchers to be able to access BAR data programmatically. We present in this update a new overarching search tool called Gaia that permits easy access to all BAR data, powered by machine learning and artificial intelligence.

## Graphical Abstract

**Graphical Abstract:**
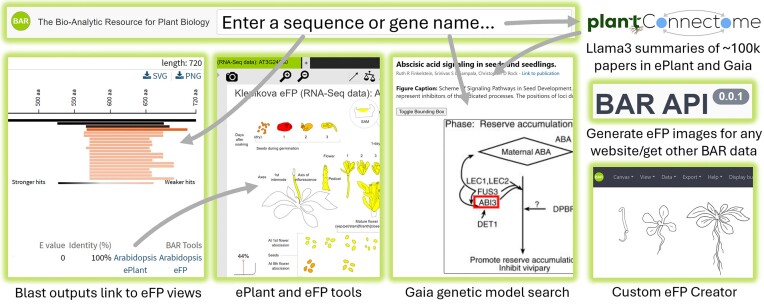

## Introduction

Online databases for plant biology, such as TAIR (1), Gramene (2), Ensembl Plants (3) and several others are now an important part of every plant researcher's toolbox and permit easy retrieval of gene annotations, sequences, homologs, gene trees and the like. The Bio-Analytic Resource for Plant Biology is one such useful ‘tool’, with strengths especially in transcriptomics and interactomics data curation and visualization. The original BAR server went online in 2005 as the ‘Botany Array Resource’ and served as a repository for the University of Toronto's Botany Department's Affymetrix microarray data and data generated by the AtGenExpress project and others. It provided three web-based tools for exploring such data—Expression Browser for performing ‘e-Northerns’, Expression Angler for undertaking coexpression analyses, and Promomer for identifying potential common *cis*-elements in the promoters of coexpressed genes (4).

Our most popular tool went live on the BAR in 2007, the ‘eFP’ Browser, or ‘electronic Fluorescent Pictograph’ Browser for pictographically exploring expression patterns in Arabidopsis (5), along with the Arabidopsis Interactions Viewer (6). The BAR was renamed at that time to the ‘Bio-Array Resource’, reflecting a change in the department's name. A Poplar eFP Browser joined the BAR in 2009 (7,8), followed by a Maize eFP Browser in 2010 (9) along with those for several other plants in the following years, with a tool permitting easy cross-species expression pattern similarity browsing, the Expressolog TreeViewer (10–23). A full list of publications describing these and other eFP views is available at https://bar.utoronto.ca/publications – these have collectively been cited more than 11 849 times, which includes around 1200 mentions of the original eFP Browser tool in papers but not the Winter *et al.* citation (Google Scholar search: *efpWeb.cgi -Winter*).

Our first ‘ePlant’ for exploring genomic data from the kilometre (variation in expression levels across geographically diverse genotypes) to nanometre (experimentally-determined and predicted protein structures) scales was released in 2011 with a complete update in 2017 (24,25). We recently incorporated Arabidopsis Alphafold2 predictions (26) into this tool, to be able to explore non-synonymous single nucleotide polymorphisms (nsSNPs) from the 1001 Genomes Project (27) in the context of these recent structural predictions. In 2013, the BAR was again renamed to the ‘Bio-Analytic Resource for Plant Biology’ (this name seems to be future-proof), at the request of its scientific advisory board, to reflect the increasing prevalence of RNA-seq data for measuring transcriptome abundance and the incorporation of many other data types. A guide to using the BAR was released in 2017 (28).

The BAR also helped to rescue/resuscitate Araport, which lost its NSF funding in 2018, by taking on the operation of ThaleMine (29,30), an Arabidopsis-specific instance of InterMine (31). Furthermore, the BAR shares its extensive collection of protein-protein interaction data with BioGRID (32)—more than 100 000 PPIs in our Arabidopsis Interactions Viewer database were manually curated with Molecular Interaction (MI) Ontology (33) terms as described in our Dong et al. publication (34). The BAR was designated a Global Core Biodata Resource by the Global Biodata Coalition in 2023. GCBRs are ‘deposition databases and knowledgebases of fundamental importance to the global life sciences and biomedical research communities, providing open access and long-term preservation of key biological data’. An overview of many BAR tools with highlights of new features is provided in Table 1.

**Table 1.** Common BAR tools and their uses. Not all tools available on the BAR are listed below

BAR tool	Short description	References
Arabidopsis eFP Browser	Visualize expression patterns for your gene of interest across 26 compendia, based on RNA-seq and ATH1-based expression data. A new feature orders compendia tabs from lowest to highest expression.	(5,35–38)^a^
Arabidopsis ePlant	Visualize data from the km to nm scale for your gene of interest, including variation in gene expression across ecotypes (39), tissue-level expression, root scRNA-seq data (40), PPIs, and protein structures including AlphaFold2 structures (26). New features include Plant Reactome pathways (41) and PlantConnectome summaries (this work).	(25)
Arabidopsis Lipid Map eFP Browser	Explore the distribution of more than 200 lipids across 14 different Arabidopsis tissues.	(42)
eFP-Seq Browser	Examine read maps of RNA-seq data from Klepikova et al. (43) and other compendia along with summarized expression levels as eFP images, to identify alternative splicing/high expression levels.	(44)
Thalemine	AnArabidopsis-specific instance of Intermine (31), resuscitated from the Araport effort (29).	(30)
Expression Browser, Expression Angler, Promomer/Cistome	A suite of tools comprising the original BAR paper for Arabidopsis for viewing expression patterns across multiple genes, identifying coexpressed genes, and predicting *cis*-elements, with updates in 2016.	(4,45)
Arabidopsis Interactions Viewer (AIV) and AIV2	Explore more than 80 000 predicted PPIs and more than 100 000 experimentally-determined PPIs from many sources from Arabidopsis, along with 2.8 million PDIs (mostly from (46). Subcellular localization from SUBA (47) is also provided	(6,34)
Poplar eFP Browser	Visualize expression patterns for poplar developmentally and in response to abiotic stress.	(7,8)
*Medicago truncatula*, Soybean, Rice, Barley, and Maize eFP Browsers	These eFP Browsers were created for the ‘expressolog’ effort to identify homologs with similar patterns of expression in equivalent tissues across species. The Maize eFP Browser was updated in 2014, 2017, 2019 and 2020; the Rice eFP Browser in 2014; and Medicago one in 2015, from listed references.	(9,10,16,48–51)
Other eFP Browsers	eFP Browsers for triticale, *Eutrema salsugineum, Camelina sativa, Brachypodium distachyon*, tomato, kiwifruit, Brassica, tung tree, kalanchoë, wheat, *Marchantia polymorpha, Euphorbia peplus* and *Cacao theobroma* have been added over time.	(11–15,17,19,20, 23,52–59)
ePlants for 15 agronomically important species	ePlants for maize, poplar, tomato, *Camelina sativa*, soybean, potato, barley, *Medicago truncatula*, eucalyptus, rice, willow, sunflower, *Cannabis sativa*, wheat and sugarcane were rolled out in 2021.	(41)
Expressolog Tree Viewer	Explore sequence similarity and expression pattern similarity for a gene of interest from one of 10 species.	(10)
AGENT	Arabidopsis Gene Regulatory Network Tool for exploring published GRNs from Arabidopsis	(60)

^a^Refers to genomics, epigenomics and transcriptomics data collectively.

In this update, we present four recent additions to the BAR to improve its usefulness to the plant research community. The Gaia search tool provides a convenient way to access the many data sets and tools available on the BAR, either by keyword/identifier or sequence search. Machine learning was also used for Gaia to flag genetic model figures from more than 65 000 Arabidopsis papers followed by the identification of gene names in these figures, to permit rapid identification of a gene of interest in genetic models from open-access literature. A second addition is the use of generative AI to provide human-readable paragraphs of around 7, 000 PlantConnectome network summaries. We also report on the BAR’s API and a Custom eFP Creator tool.

## Materials and methods

The Provart Lab has worked collaboratively with researchers around the world to develop the 157 ‘electronic fluorescent pictograph’ views available in eFP Browsers and ePlant in the BAR. The data underlying this substantial visual annotation effort and other tools are stored in more than 200 relational databases holding 186 GB of data. The BAR was recently upgraded to a Dell PowerEdge R760 server.

### Gaia

The Gaia web tool was developed using the JavaScript Node runtime environment and tested using GitHub's Node.js CI workflow to be compatible with Node version 15 and up. The primary JavaScript framework used for the development of GAIA was React.

For Gaia to be fully functional, some work was developed prior to and during the development of Gaia. This includes developing the ‘ePlant Plant eFP’ as a tissue expression web widget, creating a machine learning (ML)/optical character recognition (OCR) model for detecting publication figures and determining which are genetic models (GeneNet), and a way to detect and suggest related genes through a data aggregation step.

### ePlant plant eFP

**Figure 1.**
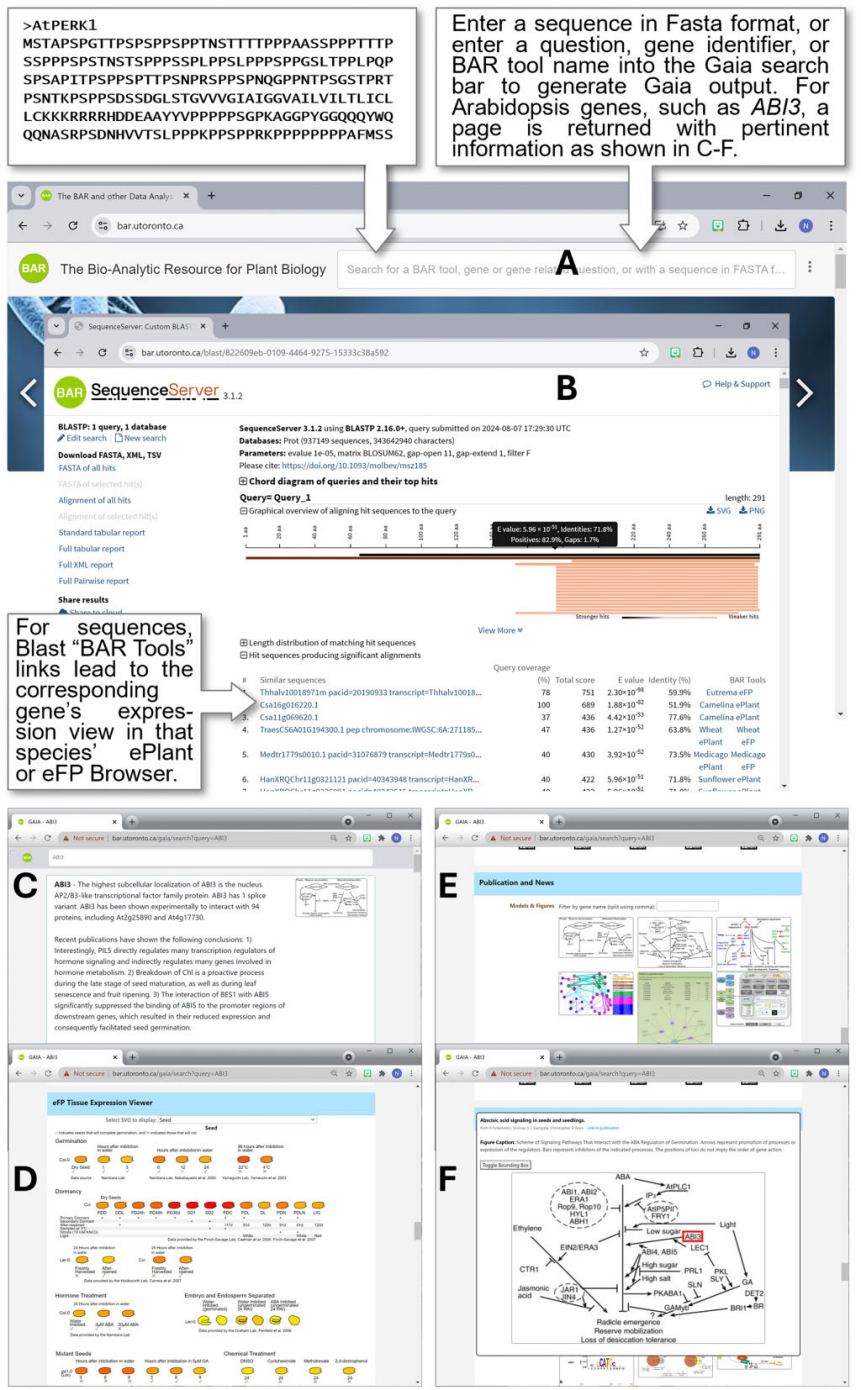
Gaia is available on the BAR’s homepage and can be searched (**A**) either with sequences in Fasta format with Blast hit outputs (**B**) linking to the respective gene's view in the corresponding species’ ePlant or eFP Browser, or by keyword search, such as gene identifier (*ABI3* is used here). In the latter case, natural language processing summaries of gene function from PubMed entries and Llama3 PlantConnectome (**C**), highest expression locations (**D**), and mentions in genetic models from the open-access literature (**E**) are provided. It is possible to have Gaia highlight the gene identifier in a given genetic model figure (a red box surrounds ABI3 in the figure shown in **F**).

The ePlant Plant eFP is an open-source web widget and modular component version of the ePlant's plant eFP viewer for *A. thaliana* (25), which can be added to any website or web application. The ePlant Plant eFP is used within Gaia to allow users to visualize tissue expression data from microarray and RNA-seq profiling experiments across 42 different compendiums. An example of the visualization can be seen in Figure 1D.

By default, if no specific compendium was pre-selected by the researcher, it will load the sample with the highest microarray or RNA-seq expression value if available (the BAR has a database of each Arabidopsis gene's highest expression level for each compendium), or, if not, load the Abiotic Stress compendium. The compendium can be changed through a dropdown which can be toggled on or off directly from the widget itself. By attaching the scripts required to load the tissue expression data visualization to the window/document, multiple instances of this tool can run across different genes with subsequent data visualizations loading from a local instance cache (expires on reload/refresh), instead of connecting to the BAR network for each call (an initial call to BAR network must happen prior to caching). This method allows developers to add an ePlant Plant eFP image to their tool and have multiple instances of the ePlant Plant eFP running simultaneously to compare data if they want while reducing network requirements. While hovering over each independent sample, such as the ePlant plant viewer, a title element for a tooltip will be displayed containing the name of the sample, expression level, control if available, sample size from experimental data and the standard deviation.

### GeneNet

GeneNet is an ML-OCR algorithm that detects which figures in a paper are genetic models and recognizes the words within the genetic model figure (61). Within Gaia, we use GeneNet to display publication model figures to the user within the executive summary (general and question) and as a UI component. Having GeneNet within Gaia allows researchers to search through published genetic models to help interpret and assist in their research to understand any possible correlation between genes present in those genetic models. The figures found by GeneNet can give researchers a visual representation of their gene and their interactions or pathways involved while giving them publication sources and details behind each figure. An example of how GeneNet is displayed in the UI can be seen in Figure 1E.

The first step includes scraping and collecting publication figures, which in the case of our initial Gaia roll-out are all from open-access publications indexed by PubMed Central (PMC) for *Arabidopsis thaliana*: 67 291 Arabidopsis papers were retrieved using NCBI’s ENTREZ API (as described in the NCBI Handbook at https://www.ncbi.nlm.nih.gov/books/NBK143764/). Next, figures present in each paper (155, 175 figures) were run through the GeneNet pipeline, which resizes all images to 600 × 600 pixels to keep input dimensions uniform. These images were processed using a Triplet Classification Network (62) that compares the figure against positive (‘is a genetic model’) and negative (‘is not a genetic model’) images to select which figures are likely genetic models. Of the 155 175 figures, GeneNet classified 11 205 as genetic models, with a success rate of around 95% (a small number of figures are protein-protein interaction networks, not genetic models). While still within the GeneNet pipeline and because standard OCR software was developed for article-like text images, object detection using Google Vision AI (https://cloud.google.com/vision) was performed to classify components in the images to identify words which can be used as searchable tags for the images, along with their x-y coordinates in the images. Once the GeneNet pipeline was complete, a post-processing step was done to determine which individual words refer to genes. This post-processing step was done by comparing all detected words >2 characters in length to those that can be found within the BAR’s Gaia's alias database (see below). Words that contain a slash within the name were searched against the whole word and the words split by the slash. For example, for the ‘GIN1/ABA2’ word in a genetic model figure, ‘GIN1/ABA2’, ‘GIN1’ and ‘ABA2’ would be used as the search term against Gaia's alias database. In total, we were able to extract 9457 unique gene terms from the genetic model figures. This pipeline permits genetic model figures which contain gene names to be easily identified by the Gaia search engine—and highlighted by a bounding box by Gaia if desired. Additionally, such figures can be further filtered for the presence of other gene names, so it is possible to identify figures that contain e.g. both ABI3 and ABI5 in them.

### Gaia data aggregation

While building Gaia, we primarily collected data from within the BAR itself, including data related to a gene's sequence, summary, structure, subcellular localization data, tissue expression data, protein interaction, DNA binding sites, Pfam domains and gene product's molecular weight. Additional data were retrieved from BioGRID (32), AraGWAS (63), KEGG (64), Phytozome (65), NCBI’s Genbank (66), UniProt (67) and TAIR (68) to help facilitate alias lookup and provide further details in Gaia's output. This information is stored in a MongoDB (https://www.mongodb.org/), along with GeneNet's figure models with their corresponding detected words, and the *x–y* coordinates of those words in the genetic model images.

### Gaia search bar

The Gaia search field on the BAR homepage allows the user to input a query consisting of a gene or a gene product, a Gene Ontology term or keyword, a BAR tool, a nucleotide or protein sequence, or a question relating to a gene or gene product. The first step in the logic flow for the search bar is to retrieve all BAR tools and to flag for which species the BAR has available data (we have generated a list of known or commonly used aliases and alternative spelling/formatting for each species name). Most data in Gaia are from Arabidopsis, although we plan on expanding Gaia for other species in the future.

The Gaia search bar also autofill suggestions. In the instance that an inputted query returns less than five results, Gaia's search bar will broaden the search to look for non-species-specific searches and include instances where the query itself is contained with a gene instead of genes that begin with the query. Upon pressing enter on the search bar, Gaia will begin processing the query and initiate a redirect. To determine what page should come next, the Gaia's search bar will first detect any BAR tools mentioned within the search query. If so, it will determine if there are any additional words along with the BAR tool itself. If there are additional words, it will compare these against the alias database to determine if any of those words are a gene alias and if so, and if the BAR tool has the ability to be dynamically linked for individual genes, redirect the user to that BAR tool with that gene loaded and, if not, just redirect that user to the BAR tool within the query. If there are no BAR tools within the query, the Gaia will next determine if the query is a nucleotide or protein sequence by detecting whether the query length is greater than 11 characters and contains exclusively only the Fasta header character (‘>’) and A, C, G or T (for nucleotides) or characters representing the 20 amino acids (for proteins), with spaces allowed. If either of those conditions is true, the sequence will be passed to the BAR’s SequenceServer (69) instance (Figure 1B), which is set up to link to respective BAR visualization tools for query sequence matches in the BAR’s sequence database. If the search query is not a BAR tool and not a nucleotide or protein sequence, then it will redirect the user to the GAIA’s search page with their query.

### PlantConnectome summaries

**Figure 2.**
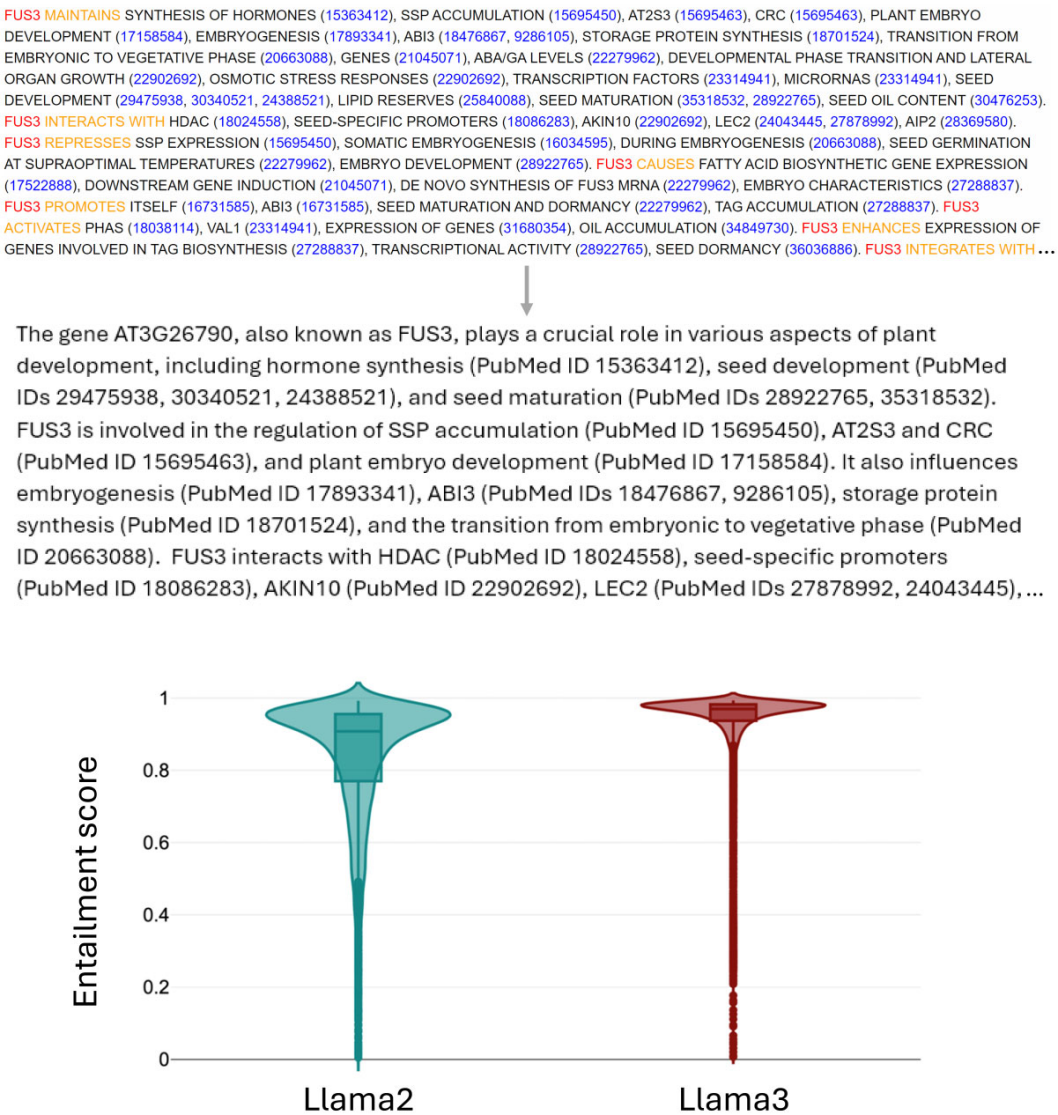
Reformatting PlantConnectome network text summaries with Llama3. Top: Partial PlantConnectome network text summary for FUS3 (At3g26790) as available on the PlantConnectome site, which we used as input into generative AI models, Llama2 and Llama3. The middle panel shows the partial output of a more human-readable Llama3 reformatted version of the inputted summary, which also maintains PubMed linkages. These Llama3 summaries are displayed in the BAR’s Gaia and ePlant tools. The bottom panel shows a comparison of BERT entailment scores across ∼7300 genes for which we could generate high-quality summaries. Higher BERT scores denote better concordance between input and output text in terms of content as determined through tokenization.

The Mutwil Lab at the Nanyang Technological University in Singapore recently used OpenAI’s GPT-3.5 Python API to extract 387 777 relationships involving genes, molecules, compartments, stresses, organs, and other plant entities from 101 341 publications (70). Each paper's abstract was submitted separately to the GPT API, which summarized the information and connections in the abstract of the paper. In this way, links back to the original publication could be maintained for referencing. The relationships were databased in an online database called PlantConnectome, which provides a knowledge graph for a given gene search term, along with a text summary of the network (top of Figure 2). In order to make this summary more human-readable and to be able to incorporate it into our ePlant and Gaia tools, we processed more than 7000 summaries available from PlantConnectome using Meta's Llama generative AI engine. We found that Llama3 had significantly improved BERT scores (71) over Llama2 when using the same prompt, with median scores of 97.0% versus 90.0%, respectively (Figure 2, bottom). We also investigated a plant-specific version of Llama, PLlama (72) for reformatting the outputs but found the results to be suboptimal. The summaries are intended to complement the curator summaries from TAIR that are also available in our ePlant and Gaia tools, and are flagged as being generated using AI.

### BAR API

**Figure 3.**
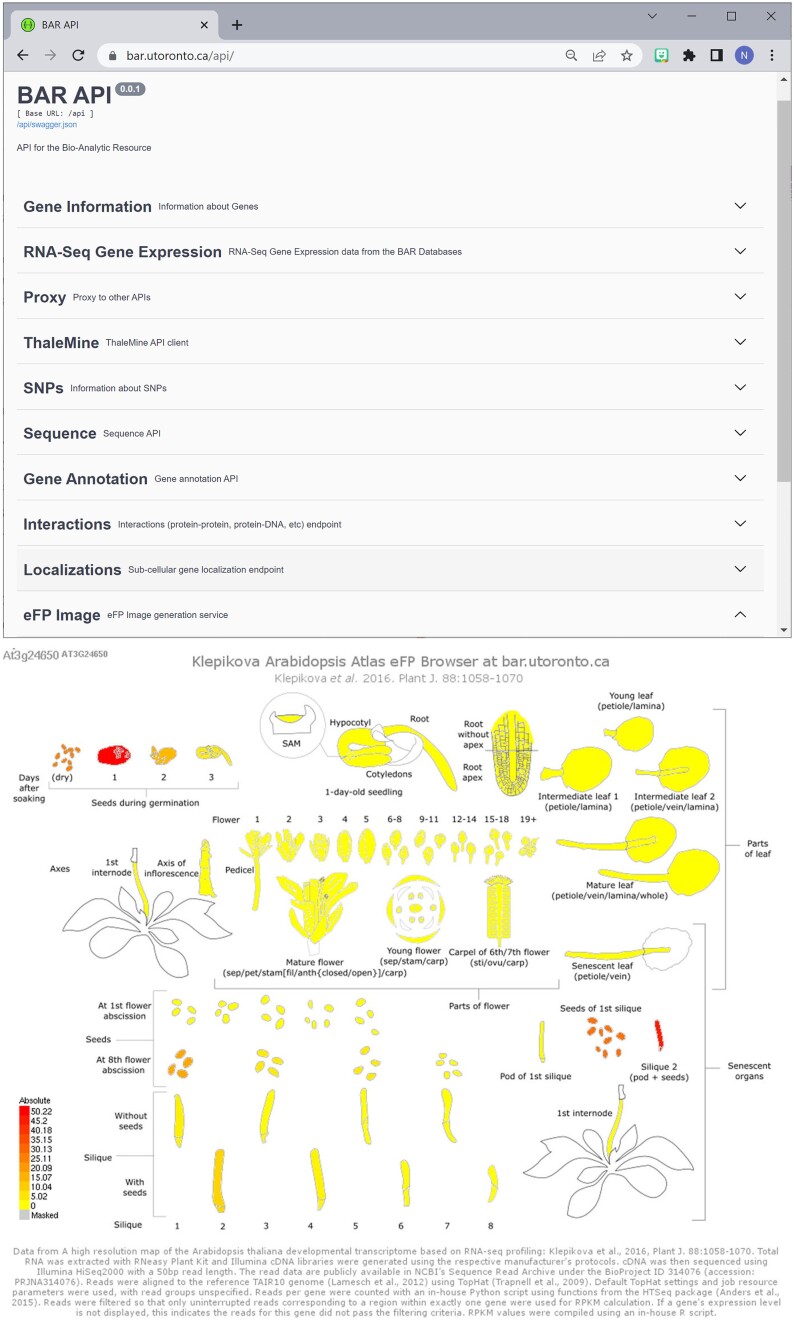
The BAR’s API page (top panel) showing different API endpoints available for researchers to be able to programmatically access BAR data sets. The eFP Image endpoint, for instance generated using https://bar.utoronto.ca/api/efp_image/efp_arabidopsis/Klepikova_Atlas/Absolute/At3g24650, will return an eFP image that can be embedded in any website on the fly (bottom image).

The original BAR was developed based on a custom-coded CGI scripts, which were written in Perl, PHP, Ruby and other languages. We have since re-written many of these using a Python Flask (https://flask.palletsprojects.com/en/3.0.x/) framework to provide API endpoints for many BAR tools. These endpoints are available to researchers to use for programmatic access to BAR visualizations and data, notably for ThaleMine data and eFP images. Several other online resources, such as TAIR, MaizeGDB, and Soybase incorporate eFP images on gene pages to pictographically display expression data. See Figure 3 for a snapshot of current API endpoints available at https://bar.utoronto.ca/api/.

### Custom eFP creator

**Figure 4.**
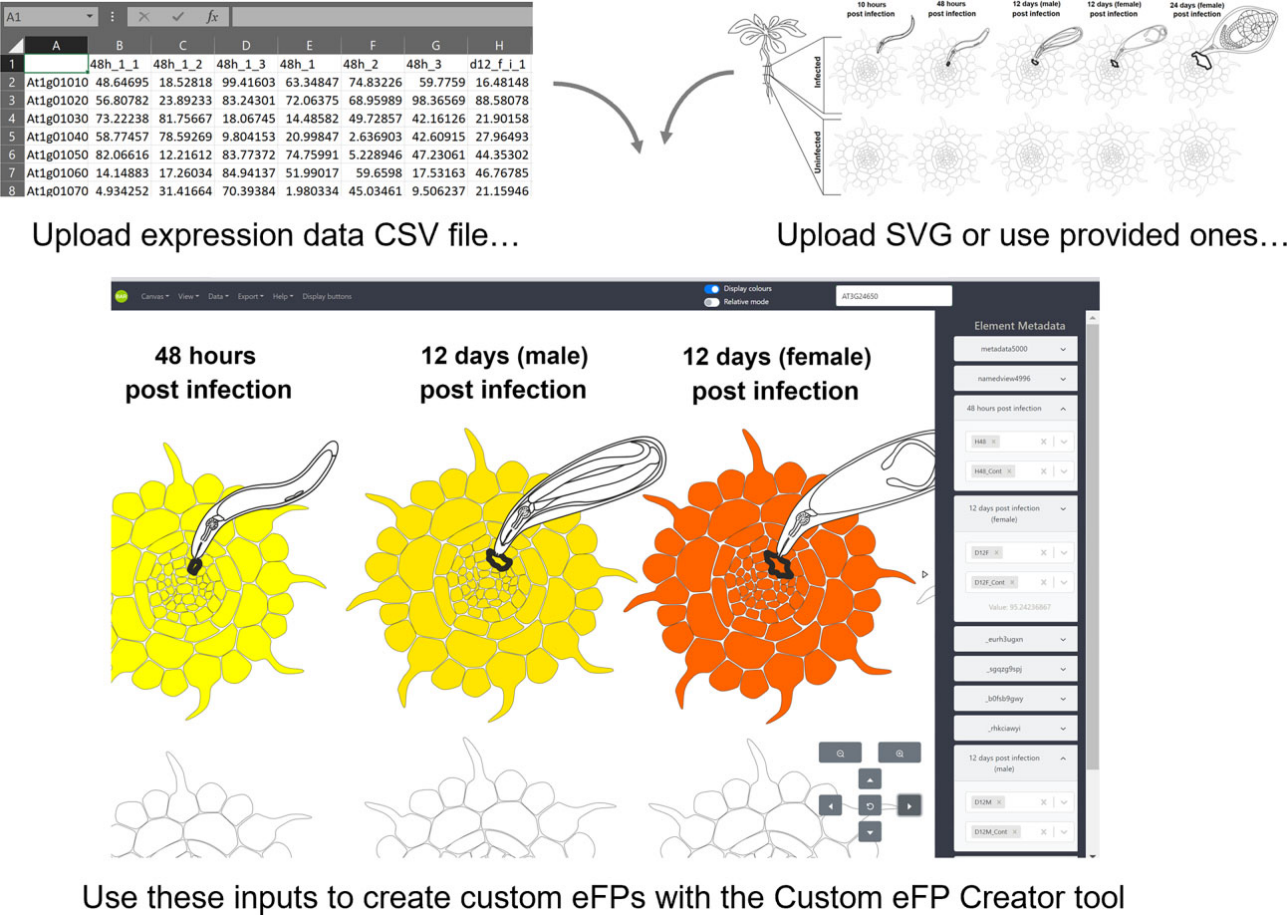
New BAR Custom eFP Creator tool. The Custom eFP Creator tool allows easy upload of expression data and SVG images to create ‘electronic fluorescent pictographs’ of gene expression (or other quantitative data) on the fly.

The most highly cited paper for BAR tools is the eFP Browser paper from 2007 (5), with around 3000 citations. The eFP Browser's ‘electronic fluorescent pictographs’ are very helpful for interpreting gene expression data because they provide a biological context, as opposed to heatmaps or tables of expression levels, where additional cognitive effort is needed to understand where the samples came from. In order to facilitate the creation of eFP images, we developed a Custom eFP Creator, which takes as input a table of gene expression values and permits the creation of eFP images with either a researcher's own SVG or using SVGs from a collection provided by us (Figure 4). This interface was programmed in Javascript.

## Results

### The AIs have It

As described in the introduction, many BAR tools have been developed over the years. Tools for visualizing gene expression information are the most popular, based on our web logs (eFP Browser, ePlant), followed by those for exploring PPI and PDIs, as possible with the BAR’s Arabidopsis Interactions Viewer 2 (34) or some ePlants (25,41). A challenge facing researchers today is an overwhelming amount of information and even finding it, notwithstanding the excellent search capabilities of Google Scholar. To make our own tools more integrated, and to help with this issue, we have followed an AI-forward approach by identifying genetic models in published papers and extracting gene identifiers from these to permit easy searching, and by providing human-readable overviews of generative AI summaries of more than 100 000 Arabidopsis papers on a gene-by-gene basis.

The genetic models in Gaia can be used to find relationships between genes in peer-reviewed publications to determine what pathways the gene of interest might be proposed to be operating in based on authors’ published models. For example, in the case of ABI3, the gene At2g20180 (*PIF1*) was highlighted as a cooperative interactor of ABI3 and also a regulator of *ABI3* expression in a figure in a review by Paik *et al.* (73). It is easily possible to identify such figures to supplement traditional search engine queries for interactions between genes of interest (Figure 1). Furthermore, the inclusion of human-readable PlantConnectome network summaries generated by Llama3 provides additional information—with traceable citations—to supplement e.g. annotator summaries from TAIR. The Llama3 summaries have a median BERT score of 97%, better than the 90% achieved when using Llama2 (Figure 2). This means that almost all information in the PlantConnectome text summaries is found in the Llama3-reformatted paragraphs we were able to generate.

### BAR API

Our ePlant and other tools make extensive use of the BAR API to retrieve information (mostly in image or JSON format) for subsequent visualization. For instance, https://bar.utoronto.ca/api/efp_image/efp_arabidopsis/Klepikova_Atlas/Absolute/At3g24650 will return an eFP image of the expression pattern of *ABI3* in the Klepikova Atlas (43), see Figure 3.

### Custom eFP creator

The Custom eFP Creator (https://bar.utoronto.ca/Custom_eFP_Creator) takes as input a CSV file with quantitative data (for example, gene expression levels) and researchers can either depict their experiments using a library of SVG images we provide (leaves, seeds, roots etc.) or upload their own SVGs with group tags uniquely labeled. The Custom eFP Creator interface permits the researcher to create the associations between sample names and group names in an easy-to-use manner and to generate an XML file that is compatible with our ePlant tools. Expression data as ‘electronic fluorescent pictographs’ may be directly visualized in the interface.

## Discussion

The BAR has come a long way from its modest start 20 years ago. We have developed 157 eFP views with collaborators around the world—most recently for *Theobroma cacao* (23), rolled out more than 15 ePlants, curated more than 100 000 PPIs, and incorporated ∼26 000 protein structures generated by Alphafold2 for exploration in the context of nucleotide polymorphism data from the 1001 Genomes Project. In this article, we expand on our efforts to make the BAR more accessible by providing direct links to our eFP Browsers and ePlants from a simple sequence search or by being able to generate a custom eFP image, and more useful, by providing AI-generated summaries from more than 100 000 papers and searchable genetic model figures covering more than 9000 genes. Future plans include working with the Plant Cell Atlas (74) consortium to incorporate single cell and single nuclei RNA-sequencing data and data from spatial transcriptomic experiments into our tools, perhaps using data feeds from the EBI’s Single Cell Expression Atlas effort (75).

## Data Availability

All BAR outputs described in this paper are open-access and are available freely without any login requirement. The BAR’s FAIRsharing page is here: https://fairsharing.org/5333, and its ThaleMine FAIRsharing page is here: https://fairsharing.org/2731. Further information about other ways of accessing BAR data may be found here: https://bar.utoronto.ca/downloads/.
